# Supplementary material for: The HDL particle composition determines its antitumor activity in pancreatic cancer
Source: Life Sci Alliance. 2022 May 16;5(9):e202101317. doi: 10.26508/lsa.202101317 (PMC9112193; doi:10.26508/lsa.202101317)
Supplement: Supplementary file 1 [file LSA-2021-01317_TableS1.docx]

**Supplementary Table S1. Murine oligonucleotide primers used for quantitative PCR and cloning of AAV-constructs.**

| Gene name | Forward primer: (5' - 3') | Reverse primer: (5' - 3') |
| --- | --- | --- |
| *Hmgcr* | CCCTCAGTTCAAATTCACAGGATG | AAGCTCTAGGACCAGCGACA |
| *Hmgcs* | GGACCGCTGCTATTCTGTCTA | TTCTAATTTAACGTCCCCAAAGGC |
| *Ldlr* | AGCCTAGAGGGGTGAACTGG | CCTGGGTGGTCAGTACAGTG |
| *SR-B1* | TGATGGAGAGCAAGCCTGTG | GCCCGTGAAGACAGTGAAGA |
| *Abca1* | CGGGTTACTATCTGACCCTGG | AGCGTGTCACTTTCATGGT |
| *Abcg1* | AGAAGGATGAAGGCAGACGA | GGGCCAGTCCTTTCATCAG |
| *Abcg4* | CAAGACCATGGCTGATGTGC | TAGAGGCAGCGCCAATCAAG |
| *Apoa1* | TCTGGTCTTCCTGACAGGGAG | TGTCTTTGACCGCATCCACA |
| *Hprt* | TCCCAGCGTCGTGATTAGC | GTGATGGCCTCCCATCTCCT |
| *Apoa1-BstB1 fwd* | TTAA**TTCGAA**ATGAAAGCTGTGGTGCTGGCCGT |  |
| *Apoa1-BsrG1 rev* | AATT**TGTACA**TCACTGGGCAGTCAGAGTCTC |  |
